# Measurement Techniques of the Magneto-Electric Coupling in Multiferroics

**DOI:** 10.3390/ma10080963

**Published:** 2017-08-17

**Authors:** M. M. Vopson, Y. K. Fetisov, G. Caruntu, G. Srinivasan

**Affiliations:** 1 School of Earth and Environmental Sciences, Faculty of Science, University of Portsmouth, Portsmouth PO1 3QL, UK; 2 Moscow Technological University, MIREA, Moscow 119454, Russia; fetisov@mirea.ru; 3 Department of Chemistry and Biochemistry, Central Michigan University, 1200 S. Franklin St., Mount Pleasant, MI 48858, USA; carun1g@cmich.edu; 4 Physics Department, Oakland University, Rochester, MI 48309-4401, USA; srinivas@oakland.edu

**Keywords:** multiferroics, magneto-electric effect, experimental techniques for multiferroics

## Abstract

The current surge of interest in multiferroic materials demands specialized measurement techniques to support multiferroics research. In this review article we detail well-established measurement techniques of the magneto-electric coupling coefficient in multiferroic materials, together with newly proposed ones. This work is intended to serve as a reference document for anyone willing to develop experimental measurement techniques of multiferroic materials.

## 1. Introduction

Multiferroic materials are defined as compounds that display at least two ferroic order states. The multiferroic phase could be formed by any possible permutations of these order states: magnetic-electric, magnetic-elastic, electric-elastic or magnetic-electric-elastic. Depending on how these phases are mixed and co-exist, typical multiferroic structures are either single-phase compounds or composites. Single-phase multiferroic materials are defined as homogenous compounds and chemically isotropic, in which electric, magnetic and possibly elastic/piezo-elastic order states coexist at any point or given location within the material. Single-phase multiferroics display extremely interesting physical properties, but they are rather limited in terms of their versatility because of their scarcity [1,2], requirement for cryogenic temperatures for their operation and reduced coupling properties [3]. For more detailed discussions of the physics and properties of single-phase multiferroic solids, we recommend a few excellent review articles on this topic [4,5]. Unlike single-phase multiferroics, composites are defined as compounds in which electric, magnetic and piezo order states coexist within the material, but they are characterized by the fact that the order phases are physically separated from each other within the multiferroic composite material. The reader can find more useful details about composite multiferroics in one of the many excellent published review articles [6,7,8,9]. This kind of multiferroic structures offers significant advantages and improvements including greater flexibility in designing specific applications [10], room-temperature operation and optimisation of the coupling properties. For these reasons, the multiferroic composites have attracted greater interest from research groups and industry going back to the 1970s [11,12,13,14]. In particular, it has been recognized that these structures potentially lead to a number of technological advances [10]. In fact, multiferroic materials are top candidates for the realization of the “universal” solid-state element that simultaneously displays magnetic, electric, elastic and tuneable optical properties within the same volume. The realization of such a material/element would lead to the creation of an “all-in-one” solid-state device capable of memory storage, logic operations, electro-mechanically and optically active, but also multifunctional electronic components that could be actively switched between capacitor, resistor, inductor, gyrator and transistor to perform multiple functions within the same solid-state element.

To facilitate the advancement of our understanding of multiferroic materials and their physical properties, there is demand for detailed specialized measurement techniques designed specifically for multiferroic materials. The majority of researchers working in the field of multiferroics have most likely migrated from the solid-state magnetism or ferroelectrics/piezo-electrics research communities, so, in most cases, they have excellent skills and capabilities in only one of the two research fields.

In this review article we aim to address this problem by reviewing the most useful measurement techniques of magneto-electric coupling in multiferroics and proposing some new ones. The article is intended to serve as a reference point for anyone, experienced or new to the field of multiferroics, who is interested in characterization techniques for multiferroic materials.

## 2. Measurement Issues

For a given multiferroic material, a typical set of experimental characterisation techniques would involve structural measurements, microscopy, dielectric/piezoelectric measurements and magnetic measurements, all under various time, temperature, mechanical and external field conditions. Indeed, these measurements are very valuable as they offer detailed information about the sample and, most importantly they can help confirm the existence of a multiferroic phase by detecting at least two co-existing ferroic order states, as defined earlier. Typically such measurements would yield magnetization (*M*), polarization (*P*), strain (*ε*), microstructure characteristics, images of ferroic domains, crystallographic structure and crystal symmetries, phase transitions, process resonances and relaxation effects, to name a few. The metrologies associated with these measurements are well known and beyond the scope of this review article.

What is of huge interest, however, are measurements of coupling properties specific to multiferroic materials, namely the “magneto-electric” coupling coefficient, which will be discussed in the next section. Unfortunately, the measurement of the magneto-electric coupling coefficient is often mistaken by the measurement of indirect evidence of magneto-electric coupling. Such measurements are important and useful, but they do not yield a numerical value of the magneto-electric coupling coefficient. In most cases they infer the presence of this coupling via a thermal measurement under the application of a large magnetic field [15], a step change in the dielectric constant at the ferroic transition temperature of the magnetic phase [16], a magnetization change due to interfacial lattice strain at structural phase transitions of the polar phase [17], a resistivity and magnetization change of magnetic phase due to interfacial lattice strain to the polar phase [18] and via optical second harmonic generation measurements [19,20], to name a few.

As already mentioned, our prime concern in this review article revolves around measurement techniques and methodologies that produce numerical values of the “magneto-electric” coupling coefficient in multiferroic materials. In the next section we briefly discuss the magneto-electric coupling coefficient, followed by the description of some useful experimental techniques used to extract this coefficient.

## 3. Magneto-Electric Coupling Coefficient

Thermodynamically it is predicted that the magneto-electric effect occurs in materials where magnetic-electric or magnetic-electric-elastic phases coexist [10]. The magneto-electric coupling facilitates the modification of electric polarization when an external magnetic field is applied, and the modification of net magnetization due to the application of an external electric field. The effect is mathematically described by the magneto-electric coupling coefficient, *α* [21]. The magneto-electric coupling coefficient can be electrically induced describing the change in the magnetic induction (*B*) of the sample due to the application of an electric field (*E*):

(1) $$\alpha_{ij}{}^{E}=\left(\frac{\partial B_{i}}{\partial E_{j}}\right)$$

or magnetically induced, describing the change in the electric polarization (*P*) due to the application of a magnetic field (*H*):

(2) $$\alpha_{ij}{}^{H}=\left(\frac{\partial P_{i}}{\partial H_{j}}\right).$$

Depending on the choice of independent thermodynamic variables, relation (1) can be also written as the partial derivative of magnetization in respect with the applied electrics field, *α^E^* = ∂M/∂E.

In any case, the true representation of magneto-electric coupling coefficient is actually in the form of a magneto-electric susceptibility second rank tensor, *α_ij_*, with nine components:

(3) $$\alpha_{ij}=\left(\begin{array}{ccc} \alpha_{11} & \alpha_{12} & \alpha_{13}\\ \alpha_{21} & \alpha_{22} & \alpha_{23}\\ \alpha_{31} & \alpha_{32} & \alpha_{33} \end{array}\right).$$

According to the Maxwell equations, the electrically and magnetically induced ME coefficients are thermodynamically equivalent [10]:

(4) $$\alpha_{ij}{}^{E}=\left(\frac{\partial B_{i}}{\partial E_{j}}\right)=\left(\frac{\partial P_{i}}{\partial H_{j}}\right)=\alpha_{ij}{}^{H}=\alpha_{ij}.$$

Although the magneto-electric tensor has nine components, in most of the studies only one of these components is non-zero. This depends on the sample/crystal symmetries, sample geometry and geometry of the applied external fields. Usually for a given experimental geometry only one of the diagonal components *α*_11_, *α*_22_, *α*_33_ or non-diagonal *α*_31_ and *α*_13_ is non-zero.

Let us now examine the electrically and magnetically induced ME coefficients:

(5) $$\alpha_{ij}=\left(\frac{\partial P_{i}}{\partial H_{j}}\right)=\varepsilon_{0}\varepsilon_{ii}\left(\frac{\partial E_{i}}{\partial H_{j}}\right),$$

where we used the relation $P_{i}=\varepsilon_{0}\chi E_{i}≅\varepsilon_{0}\varepsilon_{ii}E_{i}$ with the approximation $\chi =\varepsilon_{ii}-1≅\varepsilon_{ii}$ for materials with dielectric constant much larger than 1. We now introduce in Equation (5) the relation $B_{j}=\mu_{0}\mu_{jj}H_{j}$, resulting in:

(6) $$\alpha_{ij}=\left(\frac{\partial P_{i}}{\partial H_{j}}\right)=\varepsilon_{0}\varepsilon_{ii}\left(\frac{\partial E_{i}}{\partial H_{j}}\right)=\varepsilon_{0}\mu_{0}\varepsilon_{ii}\mu_{jj}\left(\frac{\partial E_{i}}{\partial B_{j}}\right).$$

Using Equation (1) in Equation (6) we obtain:

(7) $$\alpha_{ij}{}^{2}=\varepsilon_{0}\mu_{0}\varepsilon_{ii}\mu_{jj}$$

or

(8) $$\alpha_{ij}=\sqrt{\varepsilon_{0}\mu_{0}\varepsilon_{ii}\mu_{jj}}.$$

Equation (8) shows that all components of the magneto-electric coupling coefficient tensor must be positive since materials with negative permittivity and permeability do not exist, while the permittivity and permeability of the vacuum are positive values. Although not showing the full derivation, in most publications Equation (8) is written in non-tensor form as:

(9) $$\alpha \leq \sqrt{\varepsilon_{0}\mu_{0}\varepsilon_{r}\mu_{r}}=\sqrt{\varepsilon \mu },$$

where *ε_r_*, *µ_r_* are the dielectric relative permittivity and relative magnetic permeability of the multiferroic, respectively. One method of deriving Equation (9) makes use of the relation linking speed of light to *ε*_0_, *µ*_0_ ($c=1/\sqrt{\varepsilon_{0}\mu_{0}}$) and refractive index n to *ε_r_*, *µ_r_* ($n=\sqrt{\varepsilon_{r}\mu_{r}}$). Equation (9) becomes:

(10) $$\alpha =\sqrt{\varepsilon_{0}\mu_{0}\varepsilon_{r}\mu_{r}}=\frac{n}{c},$$

which, again, imposes only positive values of the magneto-electric coupling and it gives the well-known SI units of the coupling coefficient *α* [s/m]_SI_. The term *n*/*c* in Equation (10) can be interpreted as the inverse velocity of propagation of electromagnetic radiation in a multiferroic medium, $\alpha =\frac{n}{c}=\frac{1}{v}$. From here we apply Einstein’s Special Relativity postulate, forcing v to be *v* ≤ *c*, so that v⋅n≤c. Dividing by *c* on both sides, we get $v\cdot \frac{n}{c}\leq 1$, which leads to Equation (9), $\alpha \leq \sqrt{\varepsilon \mu }$. Therefore, the magneto-electric coupling coefficient can only take positive values in the interval $\alpha \in (0,\sqrt{\varepsilon \mu }]$. It is important to specify that the above formalism is valid for single-phase multiferroic materials. There are indeed reports of negative coupling values, but this is the case for composite multiferroics in which the magneto-electric coupling is strain/elastically mediated, and negative coupling values are allowed.

A closer inspection indicates the possibility to further manipulate Equation (5). If *V* is the voltage and *t* is the thickness of the dielectric structure, since *E* = *V*/*t*, the following relation for the magnetically induced magneto-electric effect is obtained:

(11) $$\alpha_{ij}{}^{H}=\left(\frac{\partial P_{i}}{\partial H_{j}}\right)=\varepsilon_{0}\varepsilon_{ii}\left(\frac{\partial E_{i}}{\partial H_{j}}\right)=\frac{\varepsilon_{0}\varepsilon_{r}}{t}\left(\frac{\partial V}{\partial H}\right)=\alpha_{V}{}^{H}\varepsilon_{0}\varepsilon_{r},$$

where *α_V_^H^* is the magnetically induced voltage magneto-electric coefficient defined as:

(12) $$\alpha_{V}{}^{H}=\left(\frac{\partial E}{\partial H}\right)=\frac{1}{t}\left(\frac{\partial V}{\partial H}\right).$$

The voltage magneto-electric coefficient is the main parameter used in analysing experimental measurements and designing various applications based on multiferroics. The relationship between the magnetically induced magneto-electric coupling coefficient and the voltage magneto-electric coefficient is: *α^H^* = *ε*_0_⋅*ε_r_*⋅*α_V_^H^* [22]. We have shown that, in SI units, *α^H^* and *α^E^* are both expressed in [s/m] units. However, the more practical voltage magneto-electric coefficient, *α_V_^H^* (see Equation (12)) is expressed as [V/A] in SI units and [V/cm⋅Oe] in CGS units, which are also utilized in most practical applications and scientific measurements [6,23]. In what follows, we will review some useful measurement techniques of the magneto-electric coupling coefficient.

## 4. Measurement of Magnetically Induced Magneto-Electric Coupling

Magnetically induced magneto-electric coupling coefficient is described by Equation (2), or alternatively by the magnetically induced voltage magneto-electric coefficient, Equation (12). Since it is much easier to measure a voltage than electric polarization, Equation (12) and the measurement of the voltage magneto-electric coefficient are preferred. Integrating Equation (12) we obtain a relationship between the voltage induced on the electrodes of a multiferroic device and the amplitude of an externally applied magnetic field:

(13) $$V=\alpha_{V}{}^{H}\cdot H\cdot t,$$

where *t* is the thickness of the ferroelectric layer in the case of laminates, or the thickness of the sample in the case of a single-phase composite, *H* is the amplitude of the externally applied magnetic field and *V* is the measured voltage. According to Equation (13), the voltage response of a multiferroic varies linearly with the amplitude of the applied magnetic field. It is important to mention that this relation is valid to both single-phase and composite multiferroics. However, the *H* field in this case is in fact an AC magnetic field and, unless one has the ability to apply large amplitude AC magnetic fields, the measurement requires the application of a DC magnetic field bias. The magnetic DC bias field couples with the AC magnetic field to produce a pseudo-piezo-magnetic linear response, which in turn modulates the electrical voltage response. The voltage magnetically induced magneto-electric coupling coefficient is determined experimentally in the following way:

(a) Bias the multiferroic sample under an optimal DC magnetic field bias;
(b) Apply an AC magnetic field of fixed frequency and amplitude at 0 or π angle, or any non-transverse direction, to the DC magnetic bias field [24];
(c) Measure the voltage response output of the multiferroic at various amplitudes of the applied AC magnetic field, at fixed DC magnetic bias and fixed frequency of the AC field;
(d) Plot the measured voltages as a function of the amplitude of the applied AC magnetic fields;
(e) From the obtained linear graph, as predicted by Equation (13), the magneto-electric coupling coefficient is determined as the slope of the graph divided by the thickness of the dielectric.

**Figure 1:**
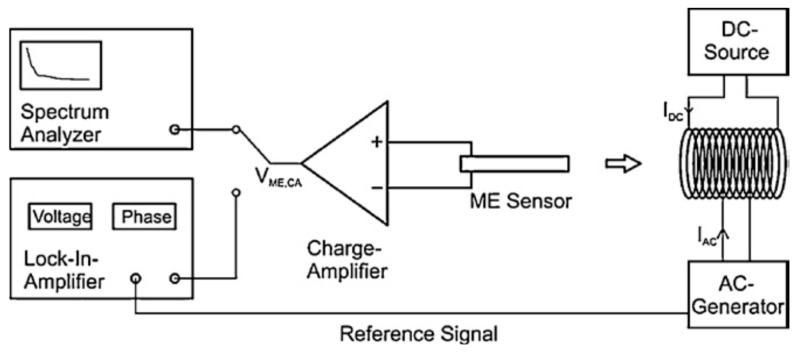
Diagram of the magnetically induced magneto-electric coupling coefficient experiment. The system allows simultaneous application of DC and AC magnetic fields, while electrically induced signals are amplified and detected to determine frequency response via a spectrum analyser or amplitude response via a lock-in amplifier. Refinements such as cryogenic or high temperature measurements and/or simultaneous applied mechanical stress are possible to implement into this generic instrument. Image source: [25].

A diagram of a typical measurement system is shown in Figure 1 (reproduced with permission from [25]):

This measurement technique is by far the most utilized in literature [26,27,28,29,30,31,32,33,34,35,36,37,38,39,40], including more sophisticated approaches where the whole measurement was performed in vacuum to reduce the acoustic air dumping effect [37]. The value of the magneto-electric coupling determined using the above method it is strongly dependent on the frequency of the applied AC magnetic field. When the frequency of the AC magnetic excitation signal matches the electro-mechanical resonance frequency of the sample, the voltage output has a sharp and enhanced resonance response. The experimental measurement of the magneto-electric coupling coefficient is therefore split into low frequency regime, far from resonance and resonance measurements. Performing a frequency sweep and analysing its response with a high-frequency impedance analyser typically determine the electro-mechanical resonance of a given sample. In either case, an optimum DC magnetic field bias must be superimposed to the AC magnetic excitation field. Following the procedure suggested above, one would naturally ask, “What is the optimum DC magnetic bias field”?

**Figure 2:**
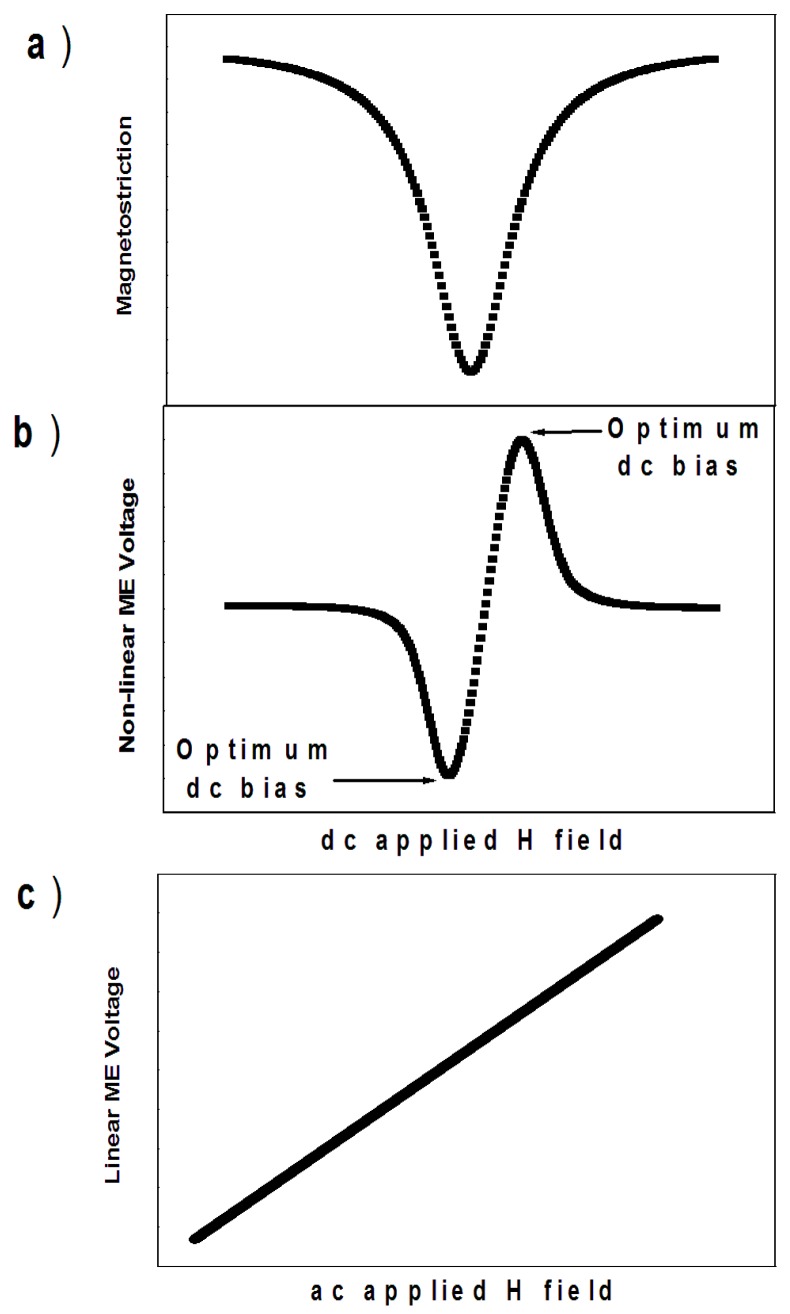
(**a**) Typical magnetostriction coefficient dependence on the DC-applied magnetic field; (**b**) typical dependence of magneto-electric induced voltage as a function of DC magnetic bias field, at constant AC magnetic field amplitude and frequency; Figure (**a**,**b**) have the same horizontal axis. (**c**) Typical dependence of magneto-electric induced voltage as a function of AC magnetic field, at constant DC magnetic bias field.

The answer is related to the fact that the voltage magneto-electric coefficient, *α_V_^H^* is a complex function of material parameters, compliances, DC magnetic bias field and frequency. This complex function has only been derived analytically for some special cases of composite multiferroics [41,42,43]. Experimental studies revealed that the magnetically induced voltage magneto-electric coefficient is highly non-linear with the DC magnetic bias field (see Figure 2b). This non-linear behaviour is related to the fact that the voltage magneto-electric coefficient depends, among other parameters, on the piezo-magnetic coefficient. The piezo-magnetic coefficient is defined as the first derivative of the magnetostriction/magnetic strain with respect to the DC magnetic field. Figure 2a shows the typical magnetic strain as a function of the DC-applied magnetic field. At zero applied magnetic fields, the strain is zero. Increasing the applied magnetic field, the strain increases rapidly and, at a given field called the saturation field, the magnetic strain becomes saturated. Beyond this point, further increases in the magnetic applied field have no effect on the sample’s strain. This saturation magnetic field roughly coincides with the saturation magnetization of the sample on the magnetic hysteresis loop. The non-linearity of the magnetic strain is transferred to the piezo-magnetic coefficient (defined as the derivative of the strain in respect with the applied field) and this in turn is transferred to the magneto-electric coupling coefficient (see Figure 2b). A typical voltage response of a multiferroic composite to applied DC magnetic fields at constant AC magnetic field amplitude and frequency is shown in Figure 2b. The optimum DC bias field corresponds to the point where the magneto-electric voltage response is maximum. This optimum DC bias field corresponds to the maximum piezo-magnetic coefficient, which in turn corresponds to the point of largest gradient in the magnetic strain–field curve (Figure 2a).

In terms of measurement of the magnetically induced magneto-electric coupling coefficient, another possible approach is to measure directly the electric polarization induced in the sample by the application of a magnetic field, Equation (2). Unlike the above technique, which involves the measurement of the open circuit induced voltage, this method involves the measurement of the short circuit-induced current, i.e., electric polarization. In this case the experimental set-up is similar to that used to measure electric polarization hysteresis loops, except that instead of applied voltages, the multiferroic sample is excited by external AC and DC magnetic fields. The easiest way to perform this experiment is to modify a commercial polarization-electric field hysteresis measurement instrument to allow the application of AC and DC magnetic fields.

## 5. Measurement of Electrically Induced Magneto-Electric Coupling

Equation (1) describes the electrically induced magneto-electric coupling, although most of the time the coefficient is expressed as $\alpha^{E}=\left(\partial M/\partial E\right)$. The measurement of this coupling coefficient simply involves a magnetic measurement of a multiferroic sample, which is subjected to applied electric fields, at zero applied magnetic field. The measurement would yield the induced magnetization in zero applied magnetic field, at different temperatures, as a function of the applied electric field/voltage. By performing this measurement at different applied electric fields/voltages, the magneto-electric coupling constant can be determined. Just as in the case of magnetically induced magneto-electric coupling, the electrically induced coupling requires either the application of an AC electric field/voltage of amplitude large enough to induce the effect, or a combination of AC and DC applied electric fields/voltages. Typically, the optimum amplitude of the AC applied electric field/voltage must be larger than the coercive field of the ferroelectric phase. Integrating relation $\alpha^{E}=\left(\partial M/\partial E\right)$, we obtain:

(14) $$M=\alpha_{E}\cdot E=\alpha_{E}\cdot V/t.$$

According to Equation (14), the electrically induced magneto-electric coupling coefficient is determined experimentally in the following way:

(a) Place the multiferroic sample in a suitable magnetometer;
(b) Under zero applied magnetic field, excite the sample with an AC electric field/voltage of fixed frequency;
(c) Measure the magnetization of the multiferroic sample at various amplitudes of the applied AC electric field/voltage;
(d) Plot the measured *M* values as a function of the amplitude of the applied AC electric field/voltage;
(e) From the obtained linear graph, as predicted by Equation (14), the magneto-electric coupling coefficient is determined as the slope of the graph if *M* = *M*(*E*), or the slope of the graph times the thickness of the dielectric if *M* = *M*(*V*) is measured;

Just as in the case of the magnetically induced coupling, there is a frequency dependence of the *α*_E_ coefficient, and the effect is expected to be largest when the frequency of the AC electric field/voltage matches the electro-mechanical resonance frequency of the sample.

The experiment can be easily performed in any magnetometer with small modifications of the sample holder in order to allow the application of a voltage to the sample’s electrodes. These instruments could be Vibrating Sample Magnetometer (VSM), Alternating Gradient Force Magnetometer (AGFM), and Superconducting Quantum Interference Device (SQUID) magnetometer, to name a few. The main disadvantage of these instruments is the complexity of addressing electrically the sample, as they are dynamically operated with the sample under physical movement, or they are utilizing delicate sample holders and detection that are affected by the addition of electrical wires, electrodes, etc.

**Figure 3:**
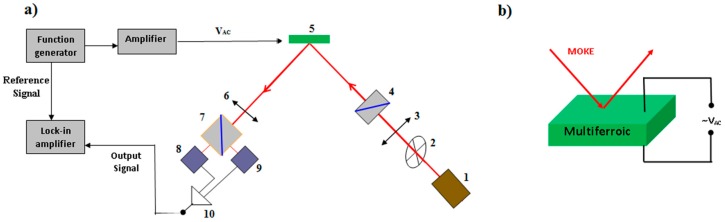
(**a**) Schematic diagram of a MOKE measurement system modified for multiferroic coupling measurements. Note the absence of the DC electromagnet. Parts of the system are: 1. CW or pulsed Laser source; 2. Beam chopper (not required if AC laser source ort if V_ac_ excitation signal is used as the reference signal for the MOKE lock-in detection; 3. *λ*/2 wave plate; 4. Polarizer; 5. Sample; 6. *λ*/4 wave plate; 7. Polarizer/Analyser; 8. Photodetector 1; 9. Photodetector 2; 10. Differential amplifier and signal output. (**b**) Diagram showing the multiferroic sample under MOKE test while subjected to electrical excitation, i.e., applied electric field.

Although with suitable modifications any magnetometer could be used to perform electrically induced magneto-electric coupling measurements, by far the most convenient way to measure this coupling is using a Magneto Optic Kerr (MOKE) magnetometer. In this technique the sample is static and the magnetization changes are detected optically, so addition of electrical wires and voltage excitations can be easily deployed. Reflection of a beam of linearly polarised light from a magnetised surface causes an orthogonal polarized component with the principal axis rotated with respect to the incoming light. The amount of rotation and ellipticity induced in the reflected beam is proportional to the magnetisation of the sample and this phenomenon is known as the magneto-optic Kerr effect (MOKE) [44,45,46]. Although the technique is extremely sensitive to very low magnetic moments, unfortunately the measured signal is not an absolute quantitative estimation of the magnetization. Hence the magnetization is measured in relative units, but the coercive fields and phase transition temperatures can be determined accurately. Figure 3 shows a schematic diagram of a simple MOKE experimental set-up for multiferroics testing.

Although there are numerous studies reporting experimental demonstration of electrically induced magnetization change [47,48], switching of the exchange bias field in ferromagnet/anti-ferromagnet structures via a voltage [49,50], electrical tuning of magnetism in hybrid spintronics/multiferroic composites [51,52,53,54] and experiments of voltage manipulation of magnetic coercive field in composite multiferroics [55,56,57,58,59], there are very few studies in which the electrically induced magneto-electric coefficient has been reported [60,61]. Moreover, it has been shown that electrically induced magnetization changes are also susceptible to measurement artefacts due to the thermal heating induced by the applied AC electric field/voltages especially at high frequencies [62]. Matsukura et al. have discussed the topic of electrical control of magnetism comprehensively in a recently published review article [63].

## 6. Measurement of Magneto-Electric Coupling from Piezo-Electric Measurements

Piezo-electric measurements involve the experimental determination of the piezo-electric coefficients or compliances of a material. These techniques are well developed and have been introduced mostly to characterize the piezo- and electro-mechanical properties of ferroelectrics utilized as mechanical actuators. Since such measurements are readily available to perform on commercial instruments, would it be possible to extract the magneto-electric coupling of a multiferroic from piezo-electric measurements? This question has been already answered [64] and the fundamentals of this technique are reviewed below.

Assuming that a multiferroic material at constant temperature contains both electric and magnetic phases, exhibiting piezo effects, piezo coupling effects and magneto-electric coupling, then using the external applied fields and stress as independent variables and following a thermodynamic formalism, it has been shown that the electric polarization of the multiferroic sample due to the application of an external stress, electric field and magnetic field is [64]:

(15) $$P_{i}=\varepsilon_{ij}E_{j}+d_{im}{}^{e}\sigma_{m}+\alpha^{eff}{}_{ij}H_{j},$$

where *P* is the electric polarization, *E* is the applied electric field, *ε* is the dielectric permittivity, *σ* is the mechanical external applied stress, *d^e^* is the piezo-electric coefficient, *α^eff^* is the effective magneto-electric coupling coefficient, H is the applied magnetic field and the above equation has been written using condensed matrix notation with *m* = 1, 2, 3, 4, 5, 6 and *i*, *j* = 1, 2, 3. Imposing short-circuit measurement conditions, so that *E* = 0, then *P* = *D*, where *D* is the electric displacement, so Equation (15) becomes:

(16) $$D_{i}=d_{im}{}^{e}\sigma_{m}+\alpha^{eff}{}_{ij}H_{j}.$$

Equation (16) gives the electric displacement of a multiferroic sample at constant temperature due to the application of an external stress (*σ*) and magnetic field (*H*). Let us remember that we assumed a hypothetical instrument capable of measuring d^e^ and we want to determine *α^eff^*. To achieve this, we rearrange Equation (16):

(17) $$d_{im}{}^{e}=\frac{D_{i}}{\sigma_{m}}-\frac{\alpha_{ij}{}^{eff}}{\sigma_{m}}H_{j}.$$

According to Equation (17), the piezo-electric coefficient of a multiferroic sample varies linearly with the applied external magnetic field. From Equation (17) we can obtain by differentiation the short circuit *α_ij_^eff^* in units of (C/m^2^⋅Oe) and the differential is the slope of the linear function (Equation (17)):

(18) $$\alpha_{ij}{}^{eff}=-\sigma_{i}\left(\frac{\partial d_{ij}{}^{e}}{\partial H_{j}}\right).$$

**Figure 4:**
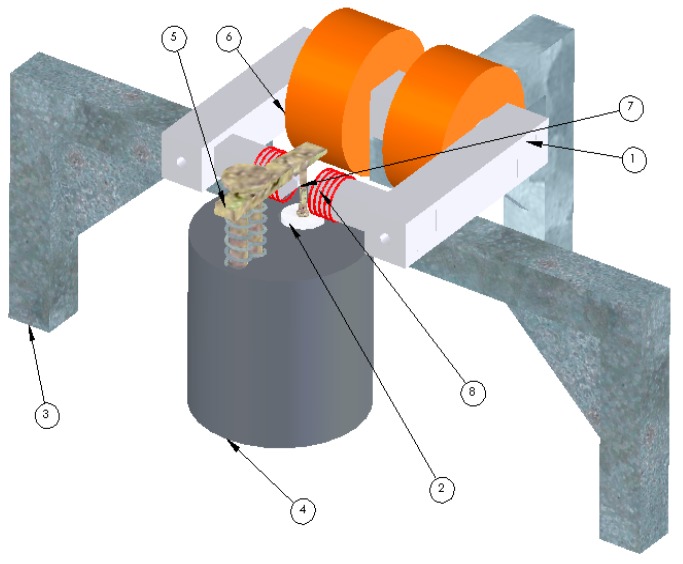
1. Electromagnet’s poles; 2. Lower contact and ac load system; 3. Electromagnet support; 4. BerlinCourt d_33_ measurement system; 5. Top sample contact; 6. DC coils of the electromagnet; 7. Sample; 8. AC coils generating the AC magnetic field. Image developed by the authors and a CAD engineer at NPL as part of the Multiprobe MET 2.1 Project.

This experiment has been successfully performed using a quasi-static piezoelectric coefficient measurement (known as the Berlincourt instrument), which has been modified to accommodate the application of DC and AC magnetic fields (see Figure 4).

The modified instrument can measure the piezoelectric coefficient and the magnetically induced ME coupling coefficient of multiferrroics. Both magnetic fields are applied in the 1 direction (i.e., in the sample plane) and they are parallel to each other, while the mechanical stress is applied to a multiferroic sample in the 3 direction. According to this measurement geometry, the *α*_31_^eff^ is determined here [64]. The DC magnetic bias is generated using a large DC electromagnet, as seen in Figure 4, although a set of permanent magnets can also be used. The AC field is produced by a set of Helmholtz coils. The magnetically induced magneto-electric coupling effect is only observed when the frequency and phase of the applied AC magnetic field match those of the AC mechanical load. In this experiment, this has been achieved by using the same function generator for the two excitations.

There are, however, other measurement techniques in which the piezo-electric coefficient is determined in open circuit conditions rather than short circuit, such as laser interferometry. Indeed, most of the magneto-electric coupling measurements are performed in open circuit conditions, with magneto-electric coupling coefficient, *α^eff^* expressed in units of V/m⋅Oe. The open circuit *α^eff^* coefficient can be determined from piezo-electric measurements by using the relationship between the short circuit piezo-electric coefficient *d^e^* and the open circuit piezo-electric coefficient *g^e^* [65]:

(19) $$d_{\mathrm{ij}}=\varepsilon_{ik}\cdot g_{kj}\approx \varepsilon_{0}\cdot \varepsilon_{r}\cdot g_{ij},$$

where *ε* is the dielectric permittivity of the material, *ε*_0_ is the permittivity of the vacuum (*ε*_0_ ≅ 8.85 × 10^−12^ C/m⋅V) and *ε_r_* is the relative dielectric constant of the material. Using Equations (18) and (19) we obtain the general expression of the open circuit magneto-electric coupling coefficient (units of V/m⋅Oe) as:

(20) $$\alpha_{ij}{}^{eff}=-\frac{\sigma_{i}}{\varepsilon_{0}\varepsilon_{r}}\left(\frac{\partial d_{ij}{}^{eff}}{\partial H_{j}}\right).$$

Equations (18) and (20) allow the elegant extraction of the magneto-electric coupling coefficient from piezo-electric measurements performed in either short circuit or open circuit conditions. This approach is very useful for multiferroic metrologies because of the rich variety of existent techniques for the measurement of the piezoelectric coefficient. According to this method, the magnetically induced magneto-electric coupling coefficient is determined experimentally in the following way:

(a) Place the multiferroic sample in a suitable piezo-electric testing instrument;
(b) The instrument must be modified to allow the simultaneous application of AC and DC magnetic fields to the sample;
(c) Measure the piezo-electric coefficient at various amplitudes of the applied AC magnetic field at fixed DC optimum bias field;
(d) Plot the piezo-electric coefficient values as a function of the amplitude of the AC magnetic field;
(e) From the slope of the linear graph, determine the magneto-electric coupling coefficient using either Equation (18) or (20), depending whether the experimental conditions are short-circuit or open-circuit.

## 7. Measurement of Magneto-Electric Coupling via Scanning Probe Microscopy

Scanning Probe Microscopy (SPM) is a generic term that defines a collection of scanning microscopy techniques all based on an atomic force microscope. There is a huge variety of possible microscopy scanning modes, but for multiferroic testing Magnetic Force Microscopy (MFM) [66], Piezo Force Microscopy (PFM) [67] and Electric Force Microscopy (EFM) [68] are the most useful techniques. Both MFM and EFM are non-contact scanning modes, while PFM is a contact measurement mode. In the PFM mode a local oscillatory electric field is generated by applying an AC voltage to a conducting tip in contact with the surface of the sample and the deformation due to the piezoelectric effect is detected by performing a standard SPM scan of the surface. In the MFM mode a two-pass method is implemented in which, during the first pass, the topography of the sample is recorded in contact mode, followed by the second pass, where the cantilever is lifted to a selected height for each scan line and scanned using the stored topography. As a result, the tip-surface separation during the second pass is kept constant. This separation must be large enough to eliminate the non-magnetic short-range surface forces and it is typically of a few tens of nanometres. Measuring the resonance frequency shift due to the magnetic tip-surface domains interaction, produces the magnetic domains profile of the sample. The EFM measurement is also a non-contact scanning mode in which the surface charge density distribution or the surface potential is imaged by applying a voltage on the tip. Just as in the case of MFM, this is a dual-pass measurement in which the standard surface topography is acquired during the first pass, and the electrostatic data is acquired during the second scan. The cantilever is driven mechanically and the electrostatic force between the biased conductive tip and the surface results in a change of the cantilever resonant frequency, which is proportional to the force gradient. In Table 1 we list the main forces involved in the measurement process of a multiferroic sample depending on the nature of the tip and the measurement mode.

*F_a_* is the total atomic repulsive force on the surface and occurs only in contact mode; *F_mag_* is the interaction force between the sample magnetization and the tip’s magnetic moment, defined for the case of a magnetic tip as:

(21) $$F_{mag}=\mu_{0}\cdot M_{tip}\cdot \nabla H_{surface}.$$

*F_e_* is the electrostatic force interaction between the charge on the tip and the surface charge distribution. This force occurs in both contact and non-contact mode if a voltage is applied to the tip:

(22) $$F_{e}=\frac{1}{2}{\left(V_{tip}-V_{surface}\right)}^{2}\frac{\partial C}{\partial z},$$

where *V_tip_* = voltage applied on the tip, *V_surface_* is the surface potential, *C* is the tip–surface capacitance and depends on the tip geometry.

*F_piezo_* is the piezo-mechanical force that occurs only in contact mode for samples that display piezo-electricity. If *x* is the piezo-electric strain, Δz the sample displacement and *z* is the sample thickness, then:

(23) $$x=\pm d_{33}\cdot E=\Delta z/z,$$

but $E\cdot z=V_{tip}$ so we can write $\Delta z=\pm d_{33}\cdot V_{tip}$. If k is the cantilever spring constant, then:

(24) $$F_{piezo}=k\cdot \Delta z=\pm d_{33}\cdot k\cdot V_{tip}.$$

*F_H_* is the interaction force between the magnetic tip and the applied external magnetic field, defined as:

(25) $$F_{H}=\mu_{0}\cdot M_{tip}\cdot \nabla H_{applied}.$$

Having defined all the dominant forces that occur in SPM measurements and having their occurrence detailed in Table 1, according to various experimental conditions, a few magneto-electric measurement options could be proposed. However, a serious challenge is to find ways to decouple the magnetic and electric forces during the non-contact measurement, or to decouple the MFM response from that of the applied external magnetic field.

**Figure 5:**
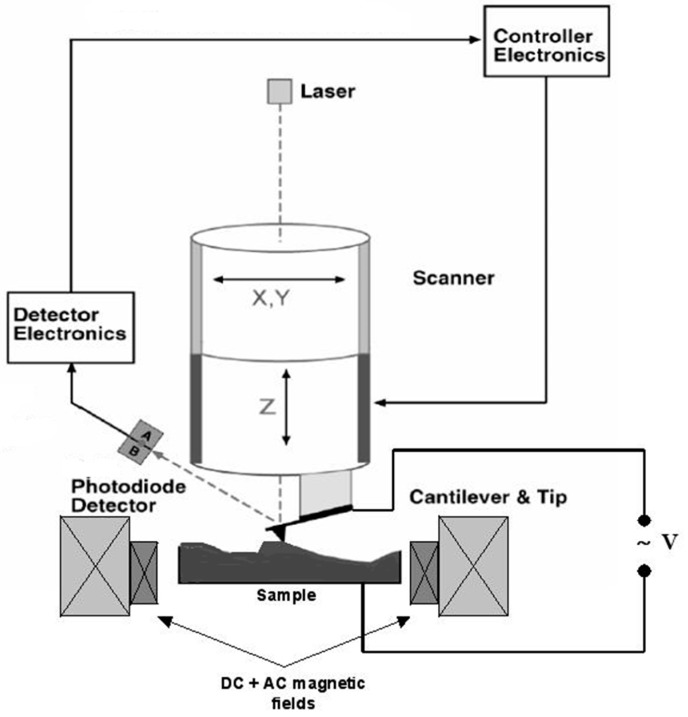
Schematic diagram of a scanning probe microscope adapted to perform multiferroic coupling measurements.

It is also important to stress that a true magneto-electric coupling measurement using SPM is in fact a static measurement where the tip is not scanning the surface but remains static. The data are acquired at a local point and the results are not images, although topography, MFM or PFM images could be acquired prior to the coupling measurement. The main benefit of the SPM technique is the ability to measure magneto-electric coupling at much reduced scales, including nanostructures. A typical measurement system could be a standard SPM modified to allow the application of AC/DC magnetic fields to the samples being tested (Figure 5), while a PFM/*d*_33_ measurement is performed. Essentially this is the technique described in Section 5, but applied at the nanoscale using an SPM. This method has already been successfully implemented [69,70], further validating the method proposed in Section 5.

## 8. Measurement of Magneto-Electric Coupling via Frequency Mixing/Conversion

Magnetically induced magneto-electric coupling discussed in Section 4 and measured at resonance frequency resulted in reports of unprecedented resolutions of magnetic field detection of a few $pT/\sqrt{Hz}$ [25,37]. At frequencies away from the resonance, the electrical noise increases while the signal decreases significantly, proportional to the Q-factor of the resonator. Therefore, operating away from resonance frequency has the effect of limiting the applicability range of such devices. Researchers at Kiel University have developed an ingenious technique to remedy this obstacle by applying a frequency mixing, also known as frequency conversion technique [37,71]. In this original method, the resonance magneto-electric coupling can be induced at arbitrary AC magnetic field excitation frequencies, away from resonance frequency. Essentially the technique involves the application of an AC magnetic field at an arbitrary frequency,

(26) $${\vec{B}}_{ac}=B_{ac}\cdot \sin\left(\omega_{ac}\cdot t\right),$$

except that instead of a DC magnetic bias field, an alternating bias field is applied. The alternating bias field is called the modulation field, *B*_mod_,

(27) $${\vec{B}}_{\mathrm{mod}}=B_{\mathrm{mod}}\cdot \sin\left(\omega_{\mathrm{mod}}\cdot t\right),$$

and it is superimposed on the AC excitation field. The method uses the non-linear characteristics of the magnetostriction curve, which changes quadratically with the magnetic field, *λ* ~ *B*^2^. The total field experienced by the sample is then:

(28) $$\vec{B}={\vec{B}}_{\mathrm{mod}}+{\vec{B}}_{ac}.$$

Assuming sinusoidal signals as described in Equations (26) and (27), the square of the total *B* field contains a product term, which expresses the frequency conversion effect:

(29) $$\begin{array}{l} {\vec{B}}^{2}=B^{2}{}_{\mathrm{mod}}+B^{2}{}_{ac}+2\cdot B_{\mathrm{mod}}\cdot B_{ac}\cdot \sin\left(\omega_{\mathrm{mod}}\cdot t\right)\cdot \sin\left(\omega_{ac}\cdot t\right)\approx \\ B_{\mathrm{mod}}\cdot B_{ac}\cdot \left(\cos((\omega_{\mathrm{mod}}-\omega_{ac})t)-\cos((\omega_{\mathrm{mod}}+\omega_{ac})t)\right) \end{array},$$

where *ω*_mod_ and *ω_ac_* are the angular frequencies of the alternating bias and the small AC signal, respectively. The fundamental effect of the application of an alternating magnetic bias field is to dynamically change the slope of the magnetostriction curve, which is seen by the small AC magnetic excitation field inducing the magneto-electric effect. In turn, the slope describes the signal transfer characteristics of the superposition of *B*_mod_ and *B_ac_* into a magnetostrictive elongation at the instantaneous operating point corresponding to the modulation frequency.

If the electro-mechanical resonance frequency of the multiferroic structure, *ω*_res_ is known, then performing the experiment so that either $\omega_{res}=\omega_{\mathrm{mod}}-\omega_{ac}$, or $\omega_{res}=\omega_{\mathrm{mod}}+\omega_{ac}$, resonant operation of the device is possible at literally arbitrary excitation frequencies. This is simply achieved by tuning the bias modulation frequency at the correct value to fulfil one of the conditions $\omega_{res}=\omega_{\mathrm{mod}}-\omega_{ac}$, or $\omega_{res}=\omega_{\mathrm{mod}}+\omega_{ac}$.

Besides offering versatility to drive the device resonantly at non-resonance excitation frequencies, this technique is also suited to wideband signals as well as non-sinusoidal signals, so various frequencies can be converted sequentially. Another significant advantage of the frequency conversion technique is the decreased sensitivity to vibration distortions at low frequencies and a reduced sensitivity against undesired acoustic crosstalk [25]. Using this technique, Jahns et al. reported a limit of detection improvement by three orders of magnitude from $\mu T/\sqrt{Hz}$ to $nT/\sqrt{Hz}$, if the detection of a low-frequency input signal outside resonance is compared with that of the same signal frequency converted to resonance using the described approach [25]. The frequency conversion/mixing technique is then used in conjunction with the method described in Section 4 to produce the numerical value of the magneto-electric coupling measurement.

## 9. Measurement of Magneto-Electric Coupling from Thermal Measurements

In 2012 a new caloric effect has been proposed, in which multiferroic materials are utilized to produce a temperature change in response to adiabatic changes of variables such as volume, strain, magnetization or electric polarization. The effect was called the multicaloric effect. Solid-state caloric effects have been known since 1917 when Weiss and Piccard made the first observation of a caloric effect in magnetic materials [72] and the effect was called the magneto-caloric effect. Today a few other solid state caloric effects have been observed including baro-caloric [73], elasto-caloric [74], giant magneto-caloric [75], electro-caloric effects [76], toroido-caloric effect [77] and oscillating caloric effect in diamagnetic materials [78,79]. However, the multicaloric effect in multiferroics has the ability to combine multiple caloric effects in response to a single adiabatic external excitation. This is possible due to particular features of multiferroic materials to accommodate multiple ferroic order states within the multiferroic solid and to display cross coupling properties between the ferroic order states. For a given multiferroic material displaying magnetic and polar order phases and magneto-electric coupling properties, the relations describing the multicaloric effect induced electrically or magnetically are [80,81,82]:

(30) $$\Delta T_{E}=-\frac{T}{C}\cdot \int_{E_{i}}^{E_{f}}\left[\frac{\alpha^{E}}{\mu_{0}\chi^{m}}\cdot {\left(\frac{\partial M}{\partial T}\right)}_{H,E}+{\left(\frac{\partial P}{\partial T}\right)}_{H,E}\right]\cdot dE$$

(31) $$\Delta T_{H}=-\frac{T}{C}\cdot \int_{H_{i}}^{H_{f}}\left[{\left(\frac{\partial M}{\partial T}\right)}_{H,E}+\frac{\alpha^{H}}{\varepsilon_{0}\chi^{e}}\cdot {\left(\frac{\partial P}{\partial T}\right)}_{H,E}\right]\cdot dH,$$

where *T* is the temperature, *C* is specific heat capacity of the system per unit volume *C* = *T* (∂*S*/∂*T*), *µ*_0_, *ε*_0_ are the magnetic permeability and dielectric permittivity of vacuum, *χ^m^* and *χ^e^* are the magnetic and electric susceptibilities, *α^E^* and *α^H^* are the electrically and magnetically induced magneto-electric coupling coefficients, *M* is magnetization and *P* is the electric polarization of the multiferroic system. A full derivation of the multicaloric effect is given in [80]. According to Equations (30) and (31), when both ∂M/∂E < 0 and ∂P/∂T < 0, a cooling effect (Δ*T_E,H_* < 0) is achieved for an adiabatic depolarisation/demagnetisation, making this effect very attractive for solid state refrigeration. However, besides solid-state refrigeration applications, the multicaloric caloric effect could also be used to develop metrologies for magneto-electric coupling coefficient estimation from thermal measurements. In order to maximize the multicaloric solid state cooling effect, a multiferroic material must have identical (or similar) ferroic phase transition temperatures to the constituent phases and the device must be operated at exactly (or close) to this transition temperature, where the partial derivatives ∂M/∂T < 0 and ∂P/∂T are maximum, i.e., *T_c_^m^* ≈ *T_c_^e^* ≈ *T*. Contrary to this requirement, a magneto-electric coupling measurement based on the multicaloric effect requires multiferroics with very different transition temperatures of their ferroic constituent phases. In this way, the different contributions to Δ*T_E,H_* in Equations (30) and (31) can be easily separated by performing the experiment at a suitable base temperature *T*. Let us make the following substitutions in Equations (30) and (31), ∂*M*/∂*T* = *γ^m^* and ∂*P*/∂*T* = *γ^e^*. Let us also assume that the transition temperature of the magnetic phase is much larger than the transition temperature of the electric phase, *T_c_^m^* > *T_c_^e^*. If the measurement is performed at an operating temperature T close to one of the transition temperatures, then depending whether *T* = *T_c_^m^* or *T* = *T_c_^e^*, either ∂*M*/∂*T* = *γ^m^* or ∂*P*/∂*T* = *γ^e^* at the operating temperature is negligible as the slope is almost zero. Since *T_c_^m^* > *T_c_^e^* then choosing the operating temperature *T* = *T_c_^e^* results in ∂*M*/∂*T* = *γ^m^* = 0 and Equations (30), (31) in integral form become:

(32) $$\Delta T_{E}=-\frac{T_{c}{}^{e}}{C}\cdot \gamma^{e}\cdot \Delta E$$

(33) $$\Delta T_{H}=-\frac{T_{c}{}^{e}}{C}\cdot \frac{\alpha^{H}}{\varepsilon_{0}\cdot \chi^{e}}\cdot \gamma^{e}\cdot \Delta H$$

Dividing Equation (33) by Equation (32), we obtain the magnetically induced magneto-electric coupling coefficient:

(34) $$\alpha^{H}=\varepsilon_{0}\cdot \chi^{e}\cdot \frac{\Delta T_{H}}{\Delta T_{E}}\cdot \frac{\Delta E}{\Delta H}$$

In a similar way, for multiferroic materials in which *T_c_^m^* < *T_c_^e^*, then choosing the operating temperature *T* = *T_c_^m^* results in ∂*P*/∂*T* = *γ^e^ =* 0 and applying the above formalism, the electrically induced magneto-electric coupling coefficient can be estimated as:

(35) $$\alpha^{E}=\mu_{0}\cdot \chi^{m}\cdot \frac{\Delta T_{E}}{\Delta T_{H}}\cdot \frac{\Delta H}{\Delta E}$$

**Figure 6:**
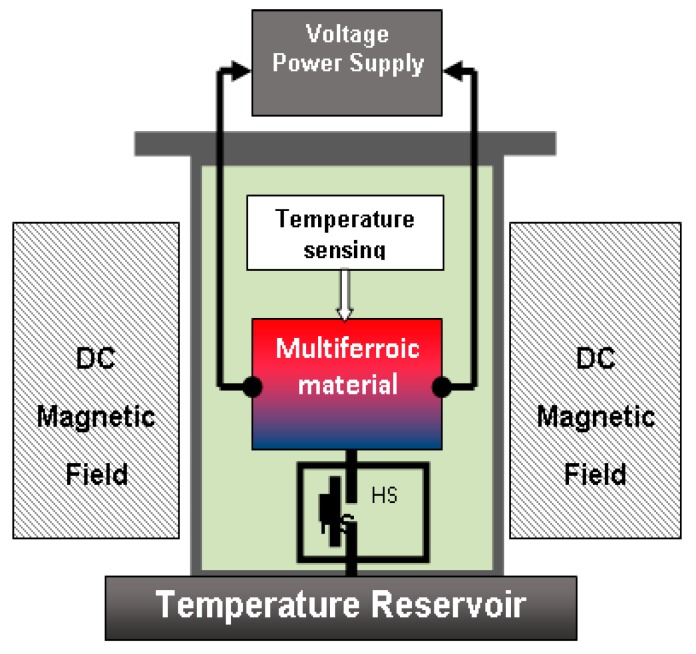
Schematic of the multicaloric testing system. HS = heat switch; Δ*T* is measured under adiabatic demagnetization and depolarization. The multiferroic material is kept adiabatically under vacuum. The HS can connect/disconnect the material to/from the temperature reservoir, providing the operating *T*. The temperature change is measured using non-contact IR thermometry or low heat capacity temperature sensors. Image modified from [83].

Remarkably, this proposed measurement procedure does not require exact knowledge of the partial derivatives ∂*M*/∂*T* = *γ^m^* or ∂*P*/∂*T* = *γ^e^* as they are reduced from the equations. One only needs to know the values of the magnetic and dielectric susceptibilities of the material under test, the values of the applied external fields, which are controlled by the experimenter and the values of the temperature change, which are measured experimentally. A possible instrument capable of measuring magneto-electric coupling coefficient from thermal measurements is shown in Figure 6, modified from [83]. According to this method, the magneto-electric coupling coefficient is determined experimentally in the following way:

(a) Place the multiferroic sample in vacuum chamber in adiabatic conditions;
(b) The instrument must be capable to apply magnetic field and electric fields to the sample, as well as to measure accurately the temperature change of the sample;
(c) A temperature reservoir can be set at a desired operating temperature and then brought in contact with the multiferroic sample via a thermal switch;
(d) Apply a large E field to the sample;
(e) While the *E* field is ON, if *T_c_^m^* > *T_c_^e^*, choose the operating temperature *T* = *T_c_^e^* and bring the sample at *T* = *T_c_^e^* via the thermal switch;
(f) Cut the thermal link to the reservoir;
(g) Reduce the *E* applied field to zero;
(h) Measure the temperature change Δ*T_E_*;
(i) Bring the sample back to the operating temperature *T* = *T_c_^e^*;
(j) Apply a large magnetic field and then bring the sample to adiabatic conditions;
(k) Reduce the applied magnetic field to zero and measure the temperature change Δ*T_H_*;
(l) Use Equation (34) to derive the magnetically induced magneto-electric coupling coefficient.
(m) If *T_c_^m^* < *T_c_^e^*, choose the operating temperature *T* = *T_c_^m^* and repeat the above procedure;
(n) Extract the electrically induced magneto-electric coupling coefficient using Equation (35).

## 10. Measurement of Non-Linear Magneto-Electric Coupling Coefficients

Linear magneto-electric effect induced magnetically involves the application of a suitable DC magnetic bias field and a small amplitude AC magnetic excitation field, as discussed in detail in Section 4. The multiferroic structure generates a magneto-electric voltage at the frequency of the excitation AC magnetic field, which is linear with the amplitude of the AC magnetic field. When the frequency of the AC magnetic field matches the electro-mechanical resonance frequency of the sample (acoustic resonance of the sample), the effect can be enhanced by a few orders of magnitude. The electro-mechanical resonance depends on the size of the sample and its stiffness coefficients, and it ranges from kHz in bulk samples to MHz and GHz in micro- or nanostructures, respectively.

Scientists at MIREA, Russia [84,85] have developed a novel multiferroics measurement technique where, instead of applying a single AC magnetic excitation field, the experiment involves the application of two collinear AC magnetic fields of different frequencies. By deploying this technique, the scientists observed interesting non-linear magneto-electric coupling effects.

Assuming that H_0_ is the DC applied magnetic field and *h*(*t*) is the total AC applied magnetic field, *h*(*t*) = *h*_1_(*t*) + *h*_2_(*t*), where *h*_1_(*t*) and *h*_2_(*t*) are simultaneously applied AC magnetic fields:

(36) $$h(t)=h_{1}\cdot \cos(2\pi \cdot f_{1}t)+h_{2}\cdot \cos(2\pi \cdot f_{2}t),$$

where *f*_1_ and *f*_2_ are the frequencies of the *h*_1_(*t*) and *h*_2_(*t*) AC magnetic fields, respectively, then the total field experienced by the sample is *H* = *H*_0_ + *h*(*t*), with *h*_1_, *h*_2_ << *H*_0_. The authors showed that the voltage generated by the multiferroic due to the application of combined magnetic fields is:

(37) $$u=A\cdot d^{e}\cdot \lambda (H),$$

where *A* is a geometrical sample factor, *d^e^* is the piezo-electric coefficient and *λ*(*H*) is the magnetostriction. Considering the non-linearity of the magnetostriction, which can be expanded into a Taylor series around *H*_0_, and using Equations (36) and (37), after some algebraic manipulation, the voltage induced due to the application of *H* = *H*_0_ + *h*_1_(*t*) + *h*_2_(*t*) is given by [84]:

(38) $$\begin{array}{l} u(t)=u^{\left(0\right)}+u_{1}{}^{\left(1\right)}\cdot \cos(2\pi \cdot f_{1}t)+u_{2}{}^{\left(1\right)}\cdot \cos(2\pi \cdot f_{2}t)+u_{1}{}^{\left(2\right)}\cdot \cos(4\pi \cdot f_{1}t)+\\ u_{2}{}^{\left(2\right)}\cdot \cos(4\pi \cdot f_{2}t)+u_{mix}\cdot \cos[2\pi \cdot (f_{1}+f_{2})t]+u_{mix}\cdot \cos[2\pi \cdot (f_{1}-f_{2})t] \end{array}.$$

The full derivation of Equation (38) can be found in [84] and shows that the experiment generates: a DC voltage component *u*^(0)^; the AC components *u*_1_^(1)^ and *u*_2_^(1)^ at the frequencies *f*_1_ and *f*_2_, respectively, due to the linear magneto-electric effect; the frequency doubling voltage components *u*_1_^(2)^ and *u*_2_^(2)^ at 2*f*_1_ and 2*f*_2_ frequencies, respectively; the frequency mixing voltage component *u_mix_* with frequencies *f*_1_ + *f*_2_ and *f*_1_ − *f*_2_, describing the non-linear mixing of magnetic fields. In this method the frequency mixing is due to AC magnetic fields of different frequencies being simultaneously applied to the multiferroic structure in addition to a DC magnetic bias field, while the Kiel frequency mixing method involves an AC magnetic field being applied simultaneously with an AC magnetic bias field, with frequencies carefully selected so that the mixed frequencies match the electro-mechanical resonance frequency of the device: *f_res_* = *f*_1_ + *f*_2_ or *f_res_* = *f*_1_ − *f*_2_. If *t_e_* is the thickness of the dielectric component, the following magneto-electric coupling coefficients can be extracted from Equation (38):

(1) The standard linear magneto-electric coupling coefficients (units of V/cm⋅Oe):
  (39) $$\begin{array}{l} \alpha_{E,1}{}^{\left(1\right)}=\frac{u_{1}{}^{\left(1\right)}}{t_{e}\cdot h_{1}}\\ \alpha_{E,2}{}^{\left(1\right)}=\frac{u_{2}{}^{\left(1\right)}}{t_{e}\cdot h_{2}} \end{array}.$$
(2) The non-linear magneto-electric coupling due to the frequency doubling voltage component (units of V/cm⋅Oe^2^):
  (40) $$\begin{array}{l} \alpha_{E,1}{}^{\left(2\right)}=\frac{u_{1}{}^{\left(2\right)}}{t_{e}\cdot h_{1}{}^{2}}\\ \alpha_{E,2}{}^{\left(2\right)}=\frac{u_{2}{}^{\left(2\right)}}{t_{e}\cdot h_{2}{}^{2}} \end{array}.$$
(3) The non-linear magneto-electric coupling due to the frequency mixing voltage component (units of V/cm⋅Oe^2^):
  (41) $$\alpha_{E}{}^{\left(mix\right)}=\frac{u_{mix}}{t_{e}\cdot h_{1}\cdot h_{2}}.$$

**Figure 7:**
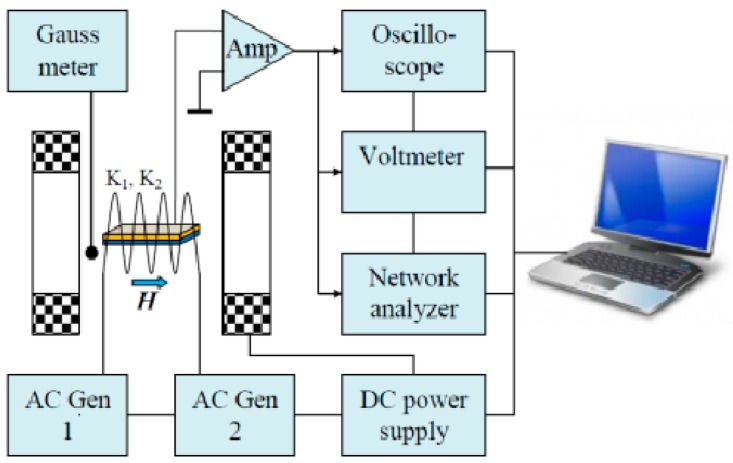
The multiferroic structure is placed in a uniform bias DC magnetic field. Alternating magnetic fields *h*_1_cos(2π*f*_1_*t*) and *h*_2_cos(2π*f*_2_*t*) with amplitudes *h*_1_, *h*_2_ and frequencies *f*_1_, *f*_2_ are created by two electromagnetic coils K_1_ and K_2_, powered by two independent generators “AC Gen1” and “AC Gen2”. Figure reprinted with permission from [85].

The block-diagram of the experimental setup is shown in Figure 7 (reprinted with permission form [85]).

## 11. Conclusions

Multiferroics are a very important class of materials exhibiting interesting multi-functional phenomena. The combination of magnetic, polar and piezo-elastic properties makes multiferroic materials very attractive for fundamental research but also technological applications. The present challenges are the discovery and manufacturing of multiferroic materials and structures that show large magneto-electric coupling at room temperature, and the development of accurate experimental characterization techniques. In order to build a clear picture of the current available metrologies for multiferroic characterization, in this review we selected some of the most relevant techniques of magneto-electric coupling measurement in multiferroics, as well as proposing new possible ones such as the extraction of the magneto-electric coupling from thermal measurements and from SPM measurements. Using this review article, a suitable measurement technique can be selected or developed depending on the structure under test, size and thermal characteristics as well as the experimental tools available to the investigator.

## Figures and Tables

**Table 1:** Forces involved in the measurement of a multiferroic sample in different configurations.

Measurement Mode		Contact SPM	Non-Contact SPM
Non-zero applied magnetic field and tip voltage	magnetic tip	*F_a_* + *F_e_* + *F_piezo_* + *F_mag_* + *F_H_*	*F_e_* + *F_mag_* + *F_H_*
	non-magnetic tip	*F_a_* + *F_e_* + *F_piezo_*	*F_e_*
Non-zero tip voltage, zero applied magnetic field	magnetic tip	*F_a_* + *F_e_* + *F_piezo_* + *F_mag_*	*F_e_* + *F_mag_*
	non-magnetic tip	*F_a_* + *F_e_* + *F_piezo_*	*F_e_*
